# Genomic Insights into and In Vitro Evaluation of Antimicrobial Combination Therapies for Carbapenem-Resistant *Acinetobacter baumannii*

**DOI:** 10.3390/medicina60071086

**Published:** 2024-07-02

**Authors:** Saadia Ijaz, Farheen Ansari, Muhammad Nawaz, Hasan Ejaz, Aftab Ahmad Anjum, Aqib Saeed, Tehreem Ali, Obaid Ur Rehman, Eeshal Fatima, Tayyaba Ijaz

**Affiliations:** 1 Institute of Molecular Biology & Biotechnology (IMBB), University of Lahore, Lahore 54590, Pakistan; 2 Institute of Microbiology, University of Veterinary and Animal Sciences, Lahore 54000, Pakistan; 3 Department of Clinical Laboratory Sciences, College of Applied Medical Sciences, Jouf University, Sakaka 72388, Saudi Arabia; 4 Department of Medicine, Services Institute of Medical Sciences, Lahore 54000, Pakistan; 5 Mayo Hospital, Health Department, Lahore 54000, Pakistan

**Keywords:** *A. baumannii*, carbapenem resistance, checkerboard method

## Abstract

*Background and Objectives: Acinetobacter baumannii* (*A. baumannii*), particularly carbapenem-resistant *A. baumannii* (CRAB), represents a grave concern in healthcare settings and is associated with high mortality. This study aimed to conduct molecular, mutational, and phylogenetic analyses of specific genes in CRAB and evaluate the synergistic effects of selected antimicrobial combinations. *Materials and Methods*: Phenotypic characterization was performed on six CRAB strains by using the Modified Hodge Test (MHT) and IMP-EDTA Double-Disc Synergy Test (IMP-EDTA DDST). Carbapenemase- and metallo-beta-lactamase-encoding genes were amplified by using Polymerase Chain Reaction. Phylogenetic analysis using the MEGA 11 tool was used to determine the evolutionary relatedness of these genes. Mutational analysis was performed by using I-Mutant, MUPro, and PHD-SNP bioinformatics tools to predict mutations in the carbapenemase-encoding genes. Microdilution checkerboard titration assessed the synergistic effects of antimicrobial combinations (azithromycin–meropenem, rifampicin–meropenem, meropenem–colistin, and azithromycin–colistin) on these CRAB isolates. *Results*: The phenotypic characterization of six CRAB isolates revealed positive results for MHT and IMP-EDTA DDST. The molecular characterization revealed that carbapenemase- and MBL-encoding genes were present in all isolates with varying frequencies, including blaOXA-51 (100%) and blaIMP (0%). The sequence analysis revealed high evolutionary relatedness to sequences in the NCBI database. The mutational analysis identified 16 mutations, of which 1 mutation (P116L) in the blaOXA-58 gene predicted a change in the protein product, potentially contributing to carbapenem resistance. The checkerboard titration method did not reveal any synergism among the tested antimicrobial combinations against CRAB. *Conclusion*: This study’s findings underscore the significant challenges posed by CRAB isolates harboring multiple resistant genes in treatment. This highlights the urgent need for novel antimicrobial agents, a crucial step towards reducing mortality rates not only in Pakistan but also globally.

## 1. Introduction

*Acinetobacter baumannii* (*A. baumannii*) is notorious for causing severe hospital-acquired infections, resulting in high mortality rates [1,2,3]. The ongoing battle against infectious diseases continues as medication resistance rapidly emerges, especially among Gram-negative bacteria [4]. Carbapenem-resistant *A. baumannii* was first reported in 1991 in the United States, and since then, *A. baumannii* species have developed significant multidrug resistance (MDR) [5].

Exploring alternative chemotherapeutic drugs effective in treating multidrug-resistant bacteria, including *A. baumannii* (MDR-*AB)*, has become a significant concern in public health. This highlights the importance of researching new and potentially beneficial compounds [4]. Clinicians actively seek alternative treatments to traditional antimicrobial agents due to the global increase in multidrug-resistant Gram-negative bacterial infections. Antimicrobials like polymyxins (colistin) are now considered viable therapeutic options to combat the shortage of new antimicrobial agents. However, it is recommended to avoid using colistin as monotherapy in cases of *A. baumannii* to prevent antimicrobial resistance development [6,7,8].

Antimicrobial resistance in *A. baumannii* arises from various mechanisms, including beta-lactamase production, enzymatic alteration of efflux pumps, the presence of aminoglycosides, deficiencies in permeability, and modifications to specific target sites [9]. Furthermore, the exploration of different mutations in resistance genes in integrons, plasmids, and transposons also contributes to the increasing resistance capability of *A. baumannii* [10]. More importantly, this resistance is thought to develop from acquired and intrinsic oxacillinases, particularly blaOXA-23 and blaOXA-51 [11,12], with blaOXA-23 being the most widespread cause of developing carbapenem resistance globally [13]. Studies indicate that insertion sequences like *ISAbaf* are crucial to developing resistance to carbapenem antimicrobials in *A. baumannii*. These insertion sequences are found upstream in the promoter region of genes associated with carbapenem resistance and contribute to enhanced expression of resistance genes [14,15]. Therefore, our objective was to develop a thorough understanding of resistance to carbapenems in *A. baumannii* by identifying mutations in these genes and whether or not they exert a deleterious effect on gene expression, inducing a subsequent change in the protein product.

The literature suggests that combining two or more antimicrobials could be a promising strategy for tackling antimicrobial resistance. These combinations of antimicrobial agents have the potential to enhance susceptibility against pathogenic bacteria, making them appealing and valuable choices for patient treatment [16]. Multiple studies have investigated the synergism of antimicrobial therapies against MDR-AB in various regions [4,6,17,18], but few studies have been observed in Pakistani scenarios. Combination therapy demonstrates the potential for broad-spectrum activity and enhanced bactericidal effects against bacterial strains [19].Hence, another aim was to assess the synergistic effects of various combination therapies against CRAB in Lahore, Pakistan. This study aimed to provide medical professionals with information about viable antimicrobial combinations to understand better how to treat patients with MDR-AB infections.

## 2. Materials and Methods

This study was conducted at the Institute of Molecular Biology and Biotechnology, University of Lahore, and the Institute of Microbiology, University of Veterinary and Animal Sciences, Lahore, from April 2021 to April 2022. The protocol of this study was approved by the ethical review committee of University of Lahore (Reg: DMB-02173003). This study is an extension of our previously published work [20]. Six carbapenemase- and metallo-beta-lactamase-producing CRAB strains, namely, S10, S67, S84, S96, S97, and S98, which are known to be resistant to the majority of antimicrobials except colistin, were selected. These strains also harbor specific carbapenemase and metallo-beta-lactamase genes, as shown in Table 1.

### 2.1. Isolation and Phenotypic Characterization of CRAB

These strains were isolated from patient samples collected from various laboratories and tertiary care facilities in Lahore, Pakistan. Biotyping was conducted through colonial morphology testing, Gram staining, biochemical assays, and API 20 E Analysis [21]. The antimicrobial resistance profiles of the *A. baumannii* isolates were assessed by using the Kirby–Bauer Method [22]. A panel of 13 antimicrobials, including ceftazidime, cefepime, piperacillin/tazobactam, doxycycline, gentamicin, tobramycin, imipenem, meropenem, ciprofloxacin, levofloxacin, amikacin, trimethoprim–sulfamethoxazole, and colistin, was employed to assess their susceptibility patterns. The MIC of colistin was determined by the broth dilution method, with interpretation guided by the Clinical and Laboratory Standards Institute [23] protocols [23]. Carbapenemase-producing *A. baumannii* strains were detected by using the MHT [24], while MBL-producing strains were identified through the Double-Disc Synergy Test (DDST) utilizing imipenem-ethylenediamine tetra acetic acid (IPM-EDTA) [25].

### 2.2. Molecular Detection of Resistant Genes

A standardized DNA extraction kit (Thermo Scientific Purification Kit for Genomic DNA; Gene JET Cat#K-0721, Waltham, MA, USA) was used to extract DNA from freshly grown *A. baumannii*. Carbapenemase-encoding genes, including blaOXA-24, blaOXA-23, blaOXA-58, and blaOXA-51, and MBL-encoding genes, including blaNDM-1, blaVIM, and blaIMP, were amplified by using forward and reverse primers used for PCR as published in our previous study [20]. The details of the primers used for PCR are also shown in Supplementary Table S1. The amplicons were sequenced, analyzed by BioEdit, and submitted to NCBI GenBank for accession numbers.

### 2.3. Phylogenetic Analysis of Carbapenemase- and MBL-Encoding Genes of CRAB

The Molecular Evolutionary Genetic Analysis version 11 (MEGA 11) tool was utilized for the phylogenetic analysis of carbapenemase-encoding genes, including the blaOXA-23, blaOXA-24, blaOXA-58, and blaOXA-51 genes. The software application aligned the study sequences with those reported in the National Center of Biotechnology Information (NCBI) by using the Clustal W-tool within MEGA 11. The initial phylogenetic tree was constructed by using a neighbor-joining (NJ) algorithm on a matrix of pairwise distances estimated through a maximum composite likelihood approach. In the phylogenetic tree, the terminal nodes represented sequences with their accession numbers connected through divergent points or internal nodes, where the genetic distances between sequences were illustrated by branch length.

### 2.4. Mutational Analysis of Carbapenemase-Encoding Genes of CRAB

A Basic Local Alignment Search Tool (BLAST) was used to convert nucleotide sequences into protein sequences, and the Fast Adaptive Shrinkage Threshold Algorithm (FASTA) sequences of proteins were retrieved. Nucleotide sequences of carbapenemase-encoding genes (i.e., the blaOXA-23, blaOXA-24, blaOXA-58, and blaOXA-51 genes) were converted into protein sequences. For mutational analysis, the I-Mutant [26], MUPro [27], and PHD-SNP [28] tools were applied. These tools were utilized to predict whether the mutations could lead to gene expression changes in protein structure and function, thereby contributing to carbapenem resistance. We classified gene expression as “Changed” if two or all three software tools predicted a potential alteration, indicating damage. Mupro and I-MUTANT assessed changes in protein stability, while PhD-SNP predicted associations with the disease based on gene expression. The effects of mutations were categorized as “Decrease”, “Increase”, or “Neutral”. In the context of I-Mutant and MUPro, an “Increase” signified that the mutation was stable and likely to impact gene expression, interpreted as a “Change” in expression. Conversely, “Decrease” indicated instability and a lower probability of altering gene expression, interpreted as “No Change”. For PHD-SNP, a stable mutation was denoted as “Disease”, indicating a “Change” in expression, whereas an unstable mutation was labeled as “Neutral”, representing “No Change” [29].

### 2.5. Combination Synergy Testing

The synergistic effects of various antimicrobial combinations were determined by using the microdilution checkerboard titration method [30]. A 96-well microplate was used to assess the synergistic effects of the antimicrobial combinations azithromycin–meropenem, rifampicin–meropenem, meropenem–colistin, and azithromycin–colistin. A table showing antibiotic concentration ranges used for the microdilution checkerboard titration method is also shown in Supplementary Table S2. All six carbapenemase- and metallo-beta-lactamase-producing CRAB strains, namely, S10, S67, S84, S96, S97, and S98, with specific genetic makeup as shown in Table 1, were exposed to these combinations. These combinations were chosen for the study based on the previous literature indicating their potential efficacy against carbapenem-resistant *A. baumannii* (CRAB) infections. The MICs of these individual antimicrobials were determined in the range of 0.25 µg/mL to 256 µg/mL by the microdilution technique following the CLSI guidelines [31]. The MIC of colistin of ≥4 µg/mL was considered resistant, and an MIC of meropenem of ≥8 µg/mL was considered resistant. No susceptibility breakpoints were available for azithromycin and rifampicin against *A. baumannii* within the CLSI guidelines. However, in this case, the CLSI criteria for staphylococci were used to determine resistance, where an MIC of ≥4 µg/mL for rifampicin was considered resistant [32]. Additionally, the CLSI criteria for Enterobacterales were used to determine resistance to azithromycin, with an MIC of ≥32 µg/mL considered resistant, as outlined by Humphries et al. in 2021 [33]. The strain ATCC 25922 of Escherichia coli was used to ensure quality control.

Each drug was diluted by using a two-fold dilution method [30]. The fractional inhibitory concentration index (FICI) was calculated as the sum of the fractional inhibitory concentration (FIC) of drug A and the FIC of drug B.

**FIC of Drug A** = MIC of drug A in combination/MIC of drug A alone

**FIC of Drug B** = MIC of drug B in combination/MIC of drug B alone

The results were interpreted based on the following criteria [34]:

- An FIC index ≤ 0.5 indicates synergy
- An FIC index within 0.5–1 indicates partial synergy
- An FIC index ≥ 1–<4 indicates indifference
- An FIC index ≥ 4 indicates antagonism.

## 3. Results

### 3.1. Phenotypic Characterization

Six *A. baumannii* isolates were identified by using API 20E. Carbapenemase production was confirmed in all six isolates by the MHT, while MBL production was detected in all isolates by using the IPM-EDTA DDST. All isolates exhibited resistance to cefepime, ceftazidime, piperacillin/tazobactam, ciprofloxacin, levofloxacin, gentamicin, amikacin, and tobramycin. However, two (33.3%) isolates showed susceptibility to doxycycline, and three (50%) isolates were susceptible to trimethoprim–sulfamethoxazole. Colistin showed effectiveness against all isolates, with none of the latter exhibiting resistance. The antimicrobial susceptibility results are summarized in Table 1.

### 3.2. Molecular Characterization of MBL- and Carbapenemase-Encoding Genes of CRAB

The blaOXA-51 gene is a naturally occurring gene unique to *A. baumannii* species, and it was detected in all six isolates. Other genes, including blaOXA-51, blaOXA-58, blaOXA-24, blaOXA-23, blaNDM-1, and blaVIM, were detected with variable frequency among the isolates of CRAB, as mentioned in Table 1. None of the isolates tested positive for blaIMP.

The DNA sequencing analysis of carbapenemase- and MBL-encoding genes of *A. baumannii* was conducted by using BioEdit, resulting in sequences for the blaOXA-24, blaOXA-23, blaOXA-58, blaOXA-51, and blaNDM-1 genes as described in our previous publication [20].

### 3.3. Phylogenetic Studies

**Figure 1:**
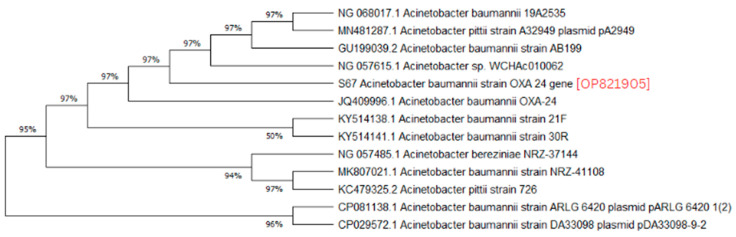
Phylogenetic analysis of blaOXA-24 gene.

**Figure 2:**
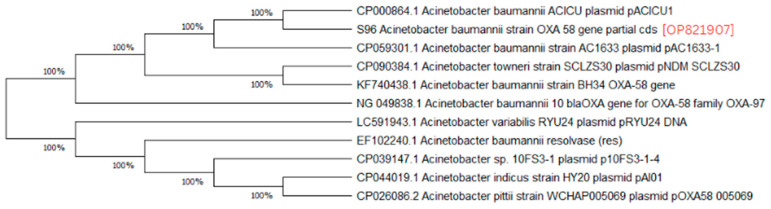
Phylogenetic analysis of blaOXA-58 gene present in study strain 96.

**Figure 3:**
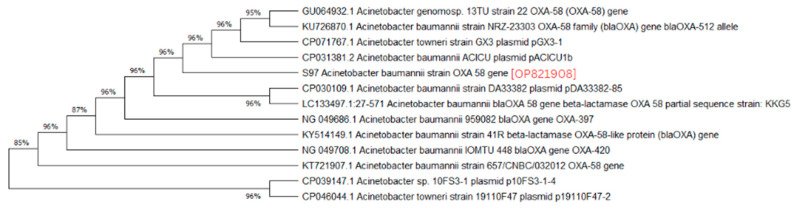
Phylogenetic analysis of blaOXA-58 gene present in study strain 97.

**Figure 4:**
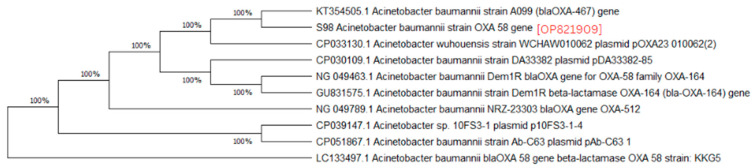
Phylogenetic analysis of blaOXA-58 gene present in study strain 98.

**Figure 5:**
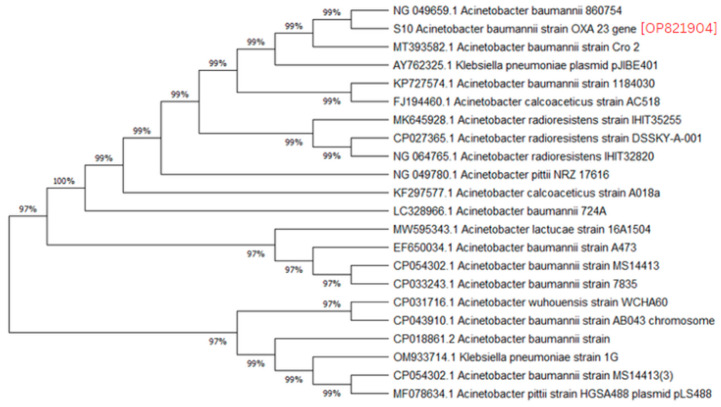
Phylogenetic analysis of blaOXA-23 gene.

**Figure 6:**
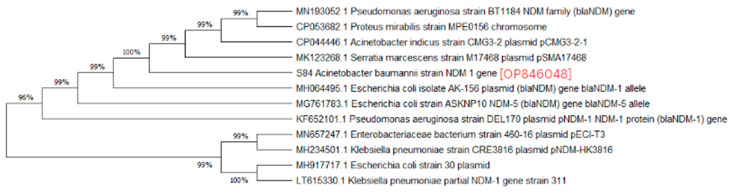
Phylogenetic analysis of blaNDM-1 gene.

**Figure 7:**
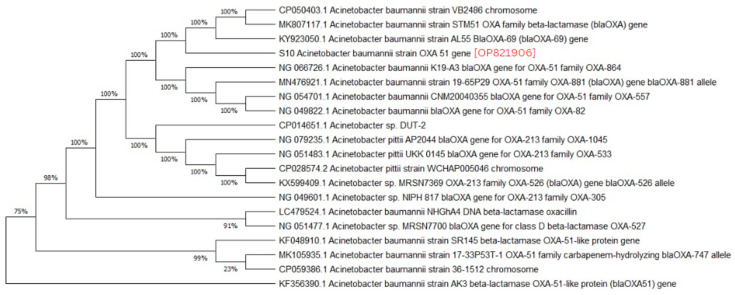
Phylogenetic analysis of blaOXA-51 gene.

A phylogenetic analysis was conducted on the DNA sequenced genes, including blaOXA-23, blaOXA-24, blaOXA-51, blaOXA-58, and blaNDM-1, from our CRAB isolates, and the results were depicted as phylogenetic trees. The respective phylogenetic trees included GenBank accession numbers for our study strains. The tree for query strain S67 OXA-24 gene in Figure 1 showed 97% evolutionary relatedness to the NCBI databases. In Figure 2, the phylogenetic representation of study strain S96 OXA-58 displayed 100% evolutionary resemblance with NCBI databases. Similarly, in Figure 3, the phylogenetic analysis of study strain S97 OXA-58 exhibited 96% evolutionary resemblance with the NCBI database. Study strain S98 OXA-58 demonstrated 100% evolutionary relatedness with the NCBI database in Figure 4. Figure 5 illustrates 99% evolutionary relatedness with the NCBI database for study strain S10 OXA-23. In Figure 6 and Figure 7, study strain S84 NDM-1 and S10 OXA-51 showed 99% and 100% evolutionary closeness with the NCBI database, respectively.

### 3.4. Mutational Analysis of blaOXA Genes of CRAB Isolates

By using I-Mutant, MUPro, and PHD-SNP software, we observed 16 mutations in the carbapenemase-encoding genes (the blaOXA-24, blaOXA-51, and blaOXA-58 genes) of the CRAB isolates, as shown in Table 2. The blaOXA-58 gene of study strain S96 had the maximum of five mutations (A60L, I59M, P116L, S121Q, and F167P); the blaOXA-51 gene of study strain S10 had two mutations (E11Q and K50N); the blaOXA-58 gene of study strain S97 had four mutations (Q2K, G4S, I61M, and A62L); the blaOXA-58 gene of study strain S98 had four mutations (A60L, I59M, S121K, and V181I); and the blaOXA-24 gene of study strain S67 had only one mutation (M69L). No mutations were observed in the blaOXA-23 gene. Table 2 also presents the amino acid changes observed at specific positions in the protein sequences of these carbapenemase-encoding genes. Table 3 predicted the impact of these 16 mutations on the genetic expression of antimicrobial resistance in the carbapenemase-encoding genes (blaOXA-24, blaOXA-51, and blaOXA-58 genes). Out of 16 mutations, only 1 (P116L) was predicted to cause a “Change” in gene expression, indicating a damaging effect. The remaining 15 mutations showed conflicting predictions regarding their impact on protein stability and subsequent gene expression, suggesting a non-damaging effect, as shown in Table 3.

### 3.5. Synergistic Effects of Antimicrobial Agents

The MIC results of all the antimicrobial agents, when used alone against all six carbapenemase and metallo-beta-lactamase producing CRAB strains, i.e., S10, S67, S84, S96, S97, and S98, with known genes, exhibited resistance to meropenem, azithromycin, and rifampicin, as described in Table 4. The MICs of colistin, rifampin, meropenem, and azithromycin were 2 µg/mL, 128 µg/mL, 64 µg/mL, and >256 µg/mL. Although the azithromycin MIC was >256, we used 256 for the FICI calculations. The checkerboard investigation with all four combinations of antimicrobials, i.e., azithromycin–meropenem, rifampicin–meropenem, meropenem–colistin, and azithromycin–colistin, showed an “indifference” result with FIC index ≥1–<4, as demonstrated in Table 5. No synergistic, partially synergistic, or antagonistic interactions were observed among the examined antimicrobial combinations.

## 4. Discussion

The study was conducted to predict the mutations in antibiotic resistance genes in CRAB and to determine the in vitro effectiveness of the different antibiotic combinations against resistant *A. baumanii*. The data on these aspects of *A. baumanii* are scarce in Pakistan. Through molecular analysis, we detected MBL- and carbapenemase-encoding genes in the CRAB isolates, focusing on seven genes: blaOXA-51, blaOXA-23, blaOXA-24, blaOXA-58, blaVIM, blaNDM-1, and blaIMP. Our results align with previous findings. A study in the UK found high prevalence of the blaNDM-1, blaIMP, blaOXA-51, and blaOXA-23 genes in 112 *A. baumannii* samples from a Lahore tertiary care setting [35]. Another UK study showed high prevalence of blaOXA-23 and blaNDM-1 genes, with lower prevalence of blaVIM and blaIMP genes [36]. Similarly, a study from Pakistan reported blaOXA-51, blaOXA-23, and blaNDM-1 as the predominant genes in their isolates [37]. Previous studies have highlighted specific gene combinations contributing to *A. baumannii* antimicrobial resistance, such as blaOXA-51, blaOXA-23, and blaVIM, which were also common in our study [38,39,40]. While blaOXA-23 and blaOXA-51 are commonly related to carbapenem resistance in *A. baumannii*, recent studies have shown a notable presence of blaNDM-1 and blaVIM, consistently with our findings [41,42,43,44].

Evolutionary relatedness among the isolates is crucial to understanding the multidrug-resistant patterns of *A. baumannii* and preventing carbapenem resistance in the community [45]. In our study, we observed a high level of evolutionary relatedness of MBL- and carbapenemase-encoding genes to the NCBI Database, with the blaOXA-51 and blaOXA-58 genes showing 100% resemblance in their sequences. In contrast, the blaOXA-23, blaOXA-24, and blaNDM-1 genes exhibited over 95% resemblance to the NCBI database. These results align with a study that found a similar high carbapenemase gene-relatedness in isolates from South Africa, a third-world country [46]. Another study conducted a phylogenetic analysis on *A. baumannii* isolates with efflux pump activity contributing to multidrug resistance. They found that all their isolates were blaOXA-51-positive and that approximately 75% exhibited efflux pump expression as a resistance mechanism. Their research identified two gene mutations, namely, the parC gene mutation and the gyrA gene mutation, responsible for inducing efflux pump expression in *A. baumannii* strains [47].

We performed mutational analysis to predict the mutations in our CRAB isolates and indirectly their potential impact on gene expression. According to our criteria, we discovered 16 mutations responsible for carbapenem resistance in our isolates; however, only one mutation predicted a notable impact on gene expression. The mutation was reported in the blaOXA-58 gene present in study strain 96 in our study, and it displayed increased stability for the mutated gene, as predicted by software. According to our criteria, the mutations reported for blaOXA-24 and blaOXA-51 were regarded as having no harmful effect on gene expression. Exploring mutations in the isolates of *A. baumannii* is a relatively novel area in research. A recent study from China reported mutations in *A. baumannii* isolates causing resistance against colistin, which is used as a “last resort” pharmacotherapy after carbapenem resistance develops in susceptible patients [48]. They reported two key mutations responsible for colistin resistance in selected isolates, thereby strengthening the argument that mutations at a genetic level drive the development of antimicrobial resistance in *A. baumannii*. The amino acid substitutions in the blaOXA-51 gene drive most of the carbapenemase activity in *A. baumannii* species. A study conducted in Hong Kong identified three key mutations that contributed to enhanced catalytic activity [49].

Additionally, we conducted antimicrobial susceptibility testing and discovered that most of our isolates were resistant to various antimicrobials. Only a limited number of isolates exhibited susceptibility to doxycycline and trimethoprim–sulfamethoxazole, while all isolates demonstrated susceptibility to colistin. Our results align with a systematic review with low resistance to doxycycline and colistin. [50]. Similarly, a study from India found that *A. baumannii* isolates exhibited susceptibility to colistin, followed by tetracyclines, in antimicrobial susceptibility testing, with most isolates demonstrating resistance to carbapenems, corroborating our findings [51].

Multiple pathways can contribute to the development of resistance against a specific class of antimicrobials [52]. Therefore, we explored the synergistic effects of various antimicrobial combinations. However, we did not observe any synergy, partial synergy, or antagonism with any combination. All the combinations, such as azithromycin–meropenem, rifampicin–meropenem, meropenem–colistin, and azithromycin–colistin, showed indifference results against all the strains (6/6, 100%). A meta-analysis of similar studies conducted in 2018 found considerable synergy rates [53] with the same combinations used in our study, contrasting with our findings. Another study in São Paulo reported that synergistic effects were observed for these combinations in colistin-susceptible isolates [6], which again contrasts with our study. A study conducted in India found a significant synergistic effect of 72% when combining colistin and meropenem against *A. baumannii* [54].

Therefore, multiple studies evaluating the synergistic effects of antimicrobial combinations reported synergism, which differed from our study, possibly due to the presence of multiple resistant genes (bla OXA-23, bla OXA-24, bla OXA-51, bla OXA-58, bla NDM-1, and bla VIM) with varying frequencies co-harboring in CRAB isolates. This high resistance in *A. baumannii* strains in our study, primarily attributed to key resistance genes, has created an urgent need to explore more suitable therapeutic agents. The inefficacy of antimicrobials against drug-resistant bacteria has sparked renewed interest in silver nanoparticles. Research has begun to investigate silver nanoparticles and other inorganic nanoparticles [55,56], which may offer innovative approaches in the face of declining antimicrobial effectiveness [57]. However, there is minimal research on this topic in Pakistan. Further research should be conducted in Pakistan to explore the efficacy of nanoparticles, both independently and in synergy with other antimicrobials, against CRAB.

There were several fundamental limitations in this study. The sample size was small. Additionally, the study was conducted by using samples from a tertiary care facility in Lahore, which may not represent the national population due to potential variations in genetic mutations among *A. baumannii* species across different regions of Pakistan. Thirdly, the study included six bacterial isolates, which may not fully encompass the diverse antibacterial phenotypic characteristics of CRAB. We only conducted phylogenetic and mutational analysis on a limited number of genes instead of performing whole-genome sequencing and analysis. Fourthly, our study only explored four antimicrobial combinations when investigating synergistic effects due to constraints such as resource limitations and the need to prioritize the most promising options based on the existing literature. Therefore, it is recommended to test more combinations, including the tetracycline group (minocycline, doxycycline, and tigecycline) and novel agents like durlobactam/sulbactam with carbapenems and colistin, against CRAB, which might produce promising results. It is further recommended to globally explore these new combinations and then formulate antibiotic stewardship policies and treatment guidelines, emphasizing the need for continuous surveillance accordingly. Future research should prioritize the whole-genome sequencing of *A. baumannii* species to understand the mutations contributing to carbapenem resistance comprehensively. Additionally, further studies should involve mutational analysis on isolates that better represent the population to elucidate trends in carbapenem resistance throughout Pakistan. Given the innovative nature of mutational analysis for carbapenem resistance in *A. baumannii* species, the scope of this research should be expanded globally, particularly in endemic regions. Mutational analysis was performed by using predictive tools, so it is recommended to incorporate experimental validation in future studies to supplement and confirm the predictions made by these computational tools.

## 5. Conclusions

In conclusion, our study demonstrated the presence of specific genes (blaOXA-23, blaOXA-24, blaOXA-51, blaOXA-58, blaNDM-1, and blaVIM) in *A. baumannii* conferring resistance to potent antimicrobials. We identified that CRAB strains carrying these genes exhibit genetic relatedness to strains found worldwide, emphasizing the need for robust preventive measures. Additionally, our research study highlighted the broad antimicrobial resistance profile of CRAB strains, with limited efficacy in combination therapies, likely due to the coexistence of multiple resistance genes. These findings suggest several future research directions, including prioritizing comprehensive whole-genome sequencing to understand carbapenem resistance in *A. baumannii* more comprehensively. We recommend expanding mutational analyses across diverse isolates in Pakistan to better represent regional resistance trends. Additionally, there is a need to globally explore and validate new antimicrobial combinations, particularly novel agents like durlobactam/sulbactam with carbapenems and colistin, to inform effective antibiotic stewardship policies and treatment guidelines. These initiatives aim to address current limitations in antimicrobial efficacy and enhance treatment strategies against CRAB infections.

## Figures and Tables

**Table 1:** Antimicrobial resistance and resistance genes detected in CRAB.

Sample ID	Source	Specimen	TZP 110 μg	FEP 30 μg	CAZ 30 μg	IPM 10 μg	MEM 10 μg	AK 30 μg	CN 10 μg	TOB 10 μg	DOX 30 μg	CIP 5 μg	LEV 5 μg	SXT 25 ug	CT *	Genes Detected	Genes Sequenced
S-10	LGH	Tracheal secretions	R	R	R	R	R	R	R	R	S	R	R	R	S	OXA-51, OXA-23, and VIM	OXA-23 and OXA-51
S-67	JHL	Tracheal secretions	R	R	R	R	R	R	R	R	R	R	R	R	S	OXA-24,OXA-23, OXA-51, and VIM	OXA-24
S-84	SHL	Blood	R	R	R	R	R	R	R	R	S	R	R	S	S	OXA-51, OXA-58, and NDM-1	NDM-1
S-96	SHL	Tracheal secretions	R	R	R	R	R	R	R	R	R	R	R	S	S	OXA-23, OXA-58, OXA-51, and VIM	OXA-58
S-97	CIP	Tracheal secretions	R	R	R	R	R	R	R	R	R	R	R	S	S	OXA-58,OXA-51, and VIM	OXA-58
S-98	CIP	Tissue	R	R	R	R	R	R	R	R	R	R	R	R	S	OXA-23, OXA-58,OXA-51, and VIM	OXA-58

LGH = Lahore General Hospital; JHL = Jinnah Hospital Lahore; SHL = Services Hospital Lahore; CIP = Chughtai Institute of Pathology. CAZ = ceftazidime, FEP = cefepime; TZP = piperacillin/tazobactam; AK = amikacin; CN = gentamicin; IPM = imipenem; MEM = meropenem; TOB = tobramycin; CIP = ciprofloxacin; DOX = doxycycline; LEV = levofloxacin; SXT = trimethoprim–sulfamethoxazole; CT = colistin; R = resistant; S = susceptible. * MIC was determined for colistin.

**Table 2:** Mutations reported in carbapenemase-encoding genes of CRAB isolates.

Strain	Gene	Mutation	Amino Acid Change	Normal Protein Sequence
S-10	blaOXA-51	E11Q	Position 11: Glutamic acid (E) replaced by Glutamine (Q)	TTTEVFKWDGEKRLFPEWEKNMTLGDAMKASAIPVYQDLARRIGLELMSKEVKRVGYGNADIGTQVDNFWLVGPLKITPQQEAQFAYKLANKTLPFSQKVQDEVQS
		K50N	Position 50: Lysine (K) replaced by Asparagine (N)	
	blaOXA-23	No mutations	No mutations	No mutations
S-67	blaOXA-24	M69L	Position 69: Methionine (M) replaced by Leucine (L)	FADDLAHNRLPFKLETQEEVKKMLLIKEVNGSKIYAKSGWGMDVTPQVGWLTGWVEQANGKKIPFSLNM
S-84	blaNDM-1	The mutational analysis was only performed on blaOXA genes		
S-96	blaOXA-58	I59M	Position 59: Isoleucine (I) replaced by Methionine (M)	TSTIPQVNNSIIDQNVQALFNEISADAVFVTYDGQNIKKYGTHLDRAKTAYIPASTFKIANALIGLENHKATSTEIFKWDGKPRFFKAWDKDFTLGEAMQASTVPVYQELARRIGPSLMQSELQRIGYGNMQIGTEVDQFWLKGPLTITPIQEVKFVYDLAQGQLPFKPEVQQQVKEMLYVERRG
		A60L	Position 60: Alanine (A) replaced by Leucine (L)	
		P116L	Position 116: Proline (P) replaced by Leucine (L)	
		S121Q	Position 121: Serine (S) replaced by Glutamine (Q)	
		F167P	Position 167: Phenylalanine (F) replaced by Proline (P)	
S-97	blaOXA-58	Q2K	Position 2: Glutamine (Q) replaced by Lysine (K)	EQTGTIPQVNNSIIDQNVQALFNEISADAVFVTYDGQNIKKYGTHLDRAKTAYIPASTFKIANALIGLENHKATSTEIFKWDGKPRFFKAWDKDFTLGEAMQASTVPVYQELARRIGPSLMQSELQRIGYGNMQIGTEVDQFWLKGPLTITPIQEVKFVYDLAQGQLPFKPEVQQQVKEMLYVERRG
		G4S	Position 4: Glycine (G) replaced by Serine (S)	
		I61M	Position 61: Isoleucine (I) replaced by Methionine (M)	
		A62L	Position 62: Alanine (A) replaced by Leucine (L)	
S-98	blaOXA-58	I59M	Position 59: Isoleucine (I) replaced by Methionine (M)	TSTIPQVNNSIIDQNVQALFNEISADAVFVTYDGQNIKKYGTHLDRAKTAYIPASTFKIANALIGLENHKATSTEIFKWDGKPRFFKAWDKDFTLGEAMQASTVPVYQELARRIGPSLMQSELQRIGYGNMQIGTEVDQFWLKGPLTITPIQEVKFVYDLAQGQLPFKPEVQQQVKEMLYV
			Position 60: Alanine (A) replaced by Leucine (L)	
		A60L	Position 121: Serine (S) replaced by Lysine (L)	
			Position 181: Valine (V) replaced by Isoleucine (I)	
		S121K		
		V181I		

Highlighted in red are amino acids usually present in the sequences that are being replaced.

**Table 3:** Mutational analysis of carbapenemase-encoding genes of CRAB isolates.

Strain	Gene	Mutation	MUpro	I-MUTANT	PhD SNP	Gene Expression
S10	OXA-51	E11Q	Decrease	Decrease	Neutral	No Change
		K50N	Decrease	Decrease	Neutral	No Change
S96	OXA-58	A60L	Decrease	Decrease	Disease	No Change
		I59M	Decrease	Decrease	Disease	No Change
		P116L	Increase	Increase	Neutral	Change
		S121Q	Decrease	Increase	Neutral	No Change
		F167P	Decrease	Decrease	Disease	No Change
S97	OXA-58	Q2K	Decrease	Decrease	Neutral	No Change
		G4S	Decrease	Decrease	Neutral	No Change
		I61M	Decrease	Decrease	Disease	No Change
		A62L	Decrease	Decrease	Disease	No Change
S98	OXA-58	A60L	Decrease	Decrease	Disease	No Change
		I59M	Decrease	Decrease	Disease	No Change
		S121K	Decrease	Increase	Neutral	No Change
		V181I	Increase	Decrease	Neutral	No Change
S67	OXA-24	M69L	Increase	Decrease	Neutral	No Change

**Table 4:** MICs of antimicrobials against selected CRAB.

Sample ID	Genes Detected	Rifampicin	Colistin	Azithromycin	Meropenem
CLSI Breakpoint		R ≥ 4 μg	R ≥ 4 μg	R ≥ 32 μg	R ≥ 8 μg
S-10	OXA-23, OXA-51, and VIM	128	2	>256	64
S-67	OXA-23, OXA-24, OXA-51, and VIM	128	2	>256	64
S-84	OXA-51, OXA-58, and NDM-1	128	2	>256	64
S-96	VIM, OXA-23, OXA-58, and OXA-51	128	2	>256	64
S-97	OXA-51, OXA-58, and VIM	128	2	>256	64
S-98	VIM, OXA-58, OXA-51, and OXA-23	128	2	>256	64

The interpretation of the MIC data involved measuring the optical density (OD) of the contents in the 96-well plate at 630 nm. CLSI = Clinical and Laboratory Standards Institute.

**Table 5:** The checkerboard test results of carbapenem-resistant *A. baumannii.*

Antimicrobial Combinations	Synergy	Partial Synergy	Indifference	Antagonism
FICI Index	≤0.5	0.5–1	≥1–<4	≥4
MEM combined with CT	-	-	S-10, S-67, S-84, S-96, S-97, and S-98	-
MEM combined with RIF	-	-	S-10, S-67, S-84, S-96, S-97, and S-98	-
MEM combined with AZM	-	-	S-10, S-67, S-84, S-96, S-97, and S-98	-
AZM combined with CT	-	-	S-10, S-67, S-84, S-96, S-97, and S-98	-

EM = meropenem; CT = colistin; RIF = rifampicin; AZM = azithromycin. FICI = fractional inhibitory concentration index.

## Data Availability

The data presented in this study are available from the corresponding author upon request.

## Supplementary Materials

The following supporting information can be downloaded at: https://www.mdpi.com/article/10.3390/medicina60071086/s1, Supplementary Table S1: Primers used in this study; Supplementary Table S2: Antimicrobial concentrations used for checkerboard assay.
