# Supplementary material for: Genomic Insights into and In Vitro Evaluation of Antimicrobial Combination Therapies for Carbapenem-Resistant Acinetobacter baumannii
Source: Medicina (Kaunas). 2024 Jul 2;60(7):1086. doi: 10.3390/medicina60071086 (PMC11278937; doi:10.3390/medicina60071086)
Supplement: Supplementary file 1 [file medicina-60-01086-s001.zip › Supplementary Table S1.pdf]

Supplementary Table S1: Primers used in this study

Gene	Primer	Reference
bla OXA-23	***Forward*** GATCGGATTGGAGAACCAGA ***Reverse*** ATTTCTGACCGCATTTCCAT	[1]
bla OXA-24	***Forward*** GGTTAGTTGGCCCCCTTAAA ***Reverse*** AGTTGAGCGAAAAGGGGATT	[1]
bla OXA-51	***Forward*** TAATGCTTTGATCGGCCTTG ***Reverse*** TGGATTGCACTTCATCTTGG	[1]
bla OXA-58	***Forward*** AAGTATTGGGGCTTGTGCTG ***Reverse*** CCCCTCTGCGCTCTACATAC	[1]
bla VIM	***Forward*** GATGGTGTTTGGTCGCATA ***Reverse*** CGAATGCGCAGCACCAG	[2]
bla IMP	***Forward*** GGAATAGAGTGGCTTAAYTCTC ***Reverse*** GGTTTAAYAAAACAACCACC	[2]
bla NDM-1	***Forward*** GGG CAG TCG CTT CCA ACG GT ***Reverse*** GTA GTG CTC AGT GTC GGC AT	[3]

1. Woodford N, Ellington MJ, Coelho JM, Turton JF, Ward ME, et al. Multiplex PCR for genes encoding prevalent OXA carbapenemases in Acinetobacter spp. International journal of antimicrobial agents, (2006); 27(4): 351-353.
2. Ellington MJ, Kistler J, Livermore DM, Woodford N. Multiplex PCR for rapid detection of genes encoding acquired metallo-β-lactamases. Journal of antimicrobial chemotherapy, (2007); 59(2): 321-322.
3. Perry JD, Naqvi SH, Mirza IA, Alizai SA, Hussain A, et al. Prevalence of faecal carriage of Enterobacteriaceae with NDM-1 carbapenemase at military hospitals in Pakistan, and evaluation of two chromogenic media. Journal of Antimicrobial Chemotherapy, (2011); 66(10): 2288-2294.
