# Supplementary material for: Genomic Insights into and In Vitro Evaluation of Antimicrobial Combination Therapies for Carbapenem-Resistant Acinetobacter baumannii
Source: Medicina (Kaunas). 2024 Jul 2;60(7):1086. doi: 10.3390/medicina60071086 (PMC11278937; doi:10.3390/medicina60071086)
Supplement: Supplementary file 1 [file medicina-60-01086-s001.zip › Supplimentary Table S2.pdf]

Supplementary Table S2: Antimicrobial concentrations used for checkerboard assay

256µg/ml of A	256 µg/ml of A<br>0.25µg/ml of B	256µg/ml of A<br>0.5µg/ml of B	256µg/ml of A<br>1µg/ml of B	256µg/ml of A<br>2µg/ml of B	256µg/ml of A<br>4µg/ml of B	256µg/ml of A<br>8µg/ml of B	256µg/ml of A<br>16µg/ml of B	256µg/ml of A<br>32µg/ml of B	256µg/ml of A<br>64µg/ml of B	256µg/ml of A<br>128µg/ml of B	512µg/ml of A<br>256µg/ml of B
128µg/ml of A	128µg/ml of A<br>0.25µg/ml of B	128µg/ml of A<br>0.5µg/ml of B	128µg/ml of A<br>1µg/ml of B	128µg/ml of A<br>2µg/ml of B	128µg/ml of A<br>4µg/ml of B	128µg/ml of A<br>8µg/ml of B	128µg/ml of A<br>16µg/ml of B	128µg/ml of A<br>32µg/ml of B	128µg/ml of A<br>64µg/ml of B	128µg/ml of A<br>128µg/ml of B	256µg/ml of A<br>256µg/ml of B
64µg/ml of A	64µg/ml of A<br>0.25µg/ml of B	64µg/ml of A<br>0.5µg/ml of B	64µg/ml of A<br>1µg/ml of B	64µg/ml of A<br>2µg/ml of B	64µg/ml of A<br>4µg/ml of B	64µg/ml of A<br>8µg/ml of B	64µg/ml of A<br>16µg/ml of B	64µg/ml of A<br>32µg/ml of B	64µg/ml of A<br>64µg/ml of B	64µg/ml of A<br>128µg/ml of B	128µg/ml of A<br>256µg/ml of B
32µg/ml of A	32µg/ml of A<br>0.25µg/ml of B	32µg/ml of A<br>0.5µg/ml of B	32µg/ml of A<br>1µg/ml of B	32µg/ml of A<br>2µg/ml of B	32µg/ml of A<br>4µg/ml of B	32µg/ml of A<br>8µg/ml of B	32µg/ml of A<br>16µg/ml of B	32µg/ml of A<br>32µg/ml of B	32µg/ml of A<br>64µg/ml of B	32µg/ml of A<br>128µg/ml of B	64µg/ml of A<br>256µg/ml of B
16µg/ml of A	16µg/ml of A<br>0.25µg/ml of B	16µg/ml of A<br>0.5µg/ml of B	16µg/ml of A<br>1µg/ml of B	16µg/ml of A<br>2µg/ml of B	16µg/ml of A<br>4µg/ml of B	16µg/ml of A<br>8µg/ml of B	16µg/ml of A<br>16µg/ml of B	16µg/ml of A<br>32µg/ml of B	16µg/ml of A<br>64µg/ml of B	16µg/ml of A<br>128µg/ml of B	32µg/ml of A<br>256µg/ml of B
8µg/ml of A	8µg/ml of A<br>0.25µg/ml of B	8µg/ml of A<br>0.5µg/ml of B	8µg/ml of A<br>1µg/ml of B	8µg/ml of A<br>2µg/ml of B	8µg/ml of A<br>4µg/ml of B	8µg/ml of A<br>8µg/ml of B	8µg/ml of A<br>16µg/ml of B	8µg/ml of A<br>32µg/ml of B	8µg/ml of A<br>64µg/ml of B	8µg/ml of A<br>128µg/ml of B	16µg/ml of A<br>256µg/ml of B
4µg/ml of A	4µg/ml of A<br>0.25µg/ml of B	4µg/ml of A<br>0.5µg/ml of B	4µg/ml of A<br>1µg/ml of B	4µg/ml of A<br>2µg/ml of B	4µg/ml of A<br>4µg/ml of B	4µg/ml of A<br>8µg/ml of B	4µg/ml of A<br>16µg/ml of B	4µg/ml of A<br>32µg/ml of B	4µg/ml of A<br>64µg/ml of B	4µg/ml of A<br>128µg/ml of B	8µg/ml of A<br>256µg/ml of B
0.125µg/ml of B	0.25µg/ml of B	0.5µg/ml of B	1µg/ml of B	2µg/ml of B	4µg/ml of B	8µg/ml of B	16µg/ml of B	32µg/ml of B	64µg/ml of B	128µg/ml of B	256µg/ml of B

A= Drug A, B= Drug B

Table displays the grid of concentrations used in a checkerboard assay, where Drug A (listed in µg/ml of A) and Drug B (listed in µg/ml of B) are combined to assess their synergistic, additive, or antagonistic effects against a microbial strain.
